# Consent for long-term storage of blood samples by Indigenous Australian research participants: the DRUID Study experience

**DOI:** 10.1186/1742-5573-4-7

**Published:** 2007-09-07

**Authors:** Joan Cunningham, Terry Dunbar

**Affiliations:** 1 Menzies School of Health Research and Institute of Advanced Studies, Charles Darwin University, PO Box 41096, Casuarina NT 0811 Australia; 2 School of Education, Faculty of Education, Health and Science, and Graduate School of Health Practice, Charles Darwin University, Darwin, Northern Territory 0909 Australia

## Abstract

**Background:**

Little is known about the characteristics of people who do and do not agree to the long-term storage and use of their biological materials, or about potential biases that may be introduced as a result of differential consent. More specifically, concerns about tissue storage and use are especially relevant among population groups for whom blood and other biological materials are culturally significant, such as Indigenous Australians. Using data from a 2003–2005 study of 1,004 Indigenous Australians, we examined participants' choices regarding long-term storage of excess blood for possible use in future studies.

**Results:**

Overall, 55% of participants agreed to long-term storage. Among 854 participants with a fasting blood sample and completed questionnaire, consent for storage was more likely among those aged 45+ years than those 15–44 (odds ratio (OR) = 1.55, 95% confidence interval (CI): 1.14, 2.11), and was similar for males and females. After adjustment for age and other covariates using logistic regression, consent was more likely for never smokers than current smokers (OR = 1.48, 95% CI: 1.04, 2.10), those reporting any non-Indigenous grandparent(s) (OR = 2.07, 95% CI: 1.50, 2.85), and those whose consent form was administered/witnessed by an Indigenous staff member (OR 1.43, 95% CI: 1.05, 1.94). Consent for long-term storage was associated with only small differences (generally less than ± 5%) in the results of assays performed on all participants' blood samples as part of the baseline health examination.

**Conclusion:**

These data show that consent for blood storage among these research participants was neither rare nor universal. It was associated with some socio-demographic/cultural factors but not with blood biochemistry. Decisions about requesting or giving consent for storage and later use of tissue samples must recognize a number of important, and potentially competing, ethical and logistical considerations.

## Background

There has been concern about declining participation in epidemiological studies, and possible reasons for this decline [1,2]. The participation of minority groups in – and their consequent ability to benefit from – research is of particular interest to researchers as well as community members [3,4]. Concurrently, researchers have continued to reflect on how best to ensure ethical practice in health research [5,6]. Ethical practice is important not only for its own sake, but also for more pragmatic reasons, such as maintaining public support for research. The level of public support can in turn affect a wide range of critical factors, from the level of government funding allocated for research to the willingness of individuals to participate in studies.

An area in which these two concerns about participation and ethical practice have converged is the collection of biological tissues from research participants and their retention in a tissue bank for later use. The nature of such future use is often unknown at the time of collection and may include potentially sensitive applications such as genetic testing [7,8]. The ethical challenges that arise, as well as the potential for reduced participation and its negative consequences, may be especially relevant among population groups for whom blood and other biological materials are culturally significant, such as Indigenous Australians [9].

Little is known about the characteristics of people who do and do not agree to the long-term storage and use of their biological materials, or about potential biases introduced as a result of differential consent. In this paper, we examine three issues using data from a study of Indigenous Australian adults: 1) the relationship between consent to store blood samples and consent for other aspects of the study; 2) the socio-demographic and cultural characteristics associated with consent to store blood samples; and 3) the likely implications of decisions regarding this consent with respect to potential for bias in future studies using stored samples.

## Methods

Data were collected in 2003–2005 as part of the DRUID Study, a study of diabetes and related conditions in urban Indigenous adults in the Darwin, Australia region. Darwin, the capital of the Northern Territory, is a tropical port city of approximately 100,000 people located on the northern coast of Australia.

The study has been described in detail elsewhere [10]. Briefly, eligible participants were aged 15 years or more, identified as Aboriginal and/or Torres Strait Islander, had lived in a defined geographic region in the Darwin area for at least 6 months, and did not live in an institutional dwelling. Eligible participants who gave consent underwent a health examination including collection of blood and urine samples, clinical and anthropometric measurements, and administration of questionnaires.

### Consent for various aspects of the study

For legal and administrative reasons, participants were asked to provide separate consent for various parts of the study by marking yes or no for each of several questions and then signing in the presence of a DRUID staff member. In addition to being asked about components of the health examination (e.g. "a fasting blood sample" or "measurement of your body size") and whether they wanted results sent to a health care provider, participants were asked to indicate what they wanted to have done with their remaining blood (and urine) samples and whether they agreed to allow study staff to access information about them from other specified sources and to contact family/friends/the participant under certain conditions (see Table 1). Missing responses were coded as non-consent.

**Table 1:** Relationship of consent to store blood with consent for other aspects of the study*. Among 1,004 urban Indigenous Australian research participants in 2003–2005.

Proportion who gave consent for study staff to:	Agreed to long-term storage (n = 550) %	Did not agreed to long-term storage (n = 454) %	Total (n = 1,004) %
Contact family and friends to help find participant	96.6	85.5	91.5
Contact participant to discuss continuation of the study beyond five years	93.1	85.9	89.8
Access information from health care provider	92.9	84.6	89.1
Access information from local Registrar of Births, Deaths and Marriages and the National Death Index	94.6	81.5	88.6
Access information from pathology services	92.4	82.2	87.8
Access information from local Department of Health and Community Services	92.2	80.4	86.8
Contact participant to discuss other related studies	88.2	73.6	81.6
Access information from the sole local private hospital	85.3	69.6	78.2
Long-term storage of excess blood samples	---	---	54.8

* *p *< 0.001 for all comparisons between those who did and did not consent.

With respect to blood samples remaining after the completion of all baseline tests that were described in the participant information sheet, participants were asked to choose one of three options:

• Destroy remaining samples;

• Store remaining samples, but contact the participant to seek permission if a researcher wishes to use them in future;

• Store remaining samples, and use them without contacting the participant, provided their use is approved by the study's Indigenous Steering Group and relevant ethics committee(s).

For the purposes of analysis, the second and third groups were combined to create a dichotomous variable: store/destroy.

**Figure 1:**
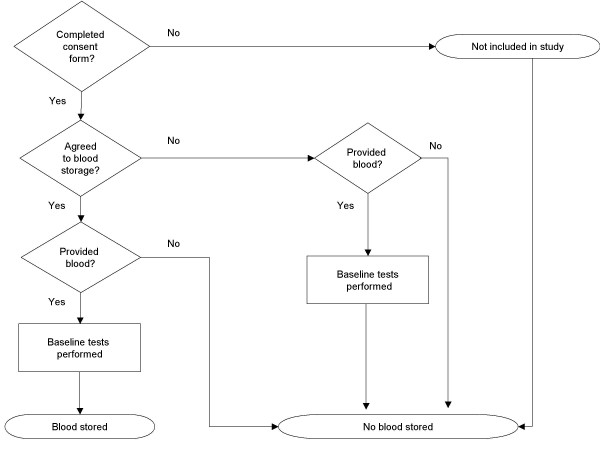
Flow chart of long-term blood storage in the DRUID Study.

As indicated in Figure 1, baseline blood tests were performed for all participants who provided blood, regardless of whether they agreed to the subsequent storage of any excess blood. All remaining blood samples from participants who did not agree to long-term storage were destroyed in late 2005. Blood samples for participants who agreed to storage are currently stored in freezers (-80C) at the Menzies School of Health Research in Darwin, under the custody of the DRUID Study Project Leader (JC). Any future use of these stored samples requires application to the Project Leader as well as approval from the Study's Management Group, its Indigenous Steering Group, the relevant human research ethics committee(s), and, for those who indicated that they wished to be contacted prior to any use, the individual participants (or if deceased, their next of kin). Should the relevant DRUID Study groups cease to exist, the custody and/or disposal of the stored samples will be determined by agreement between the Project Leader and the Chair of the Indigenous Steering Group (TD), in consultation with the relevant ethics committee(s).

### Demographic, socioeconomic and cultural variables

Available demographic information included age, sex and Indigenous status (whether identifies as Aboriginal only, Torres Strait Islander only, or both Aboriginal and Torres Strait Islander). Age was collected in whole years, and categorised for the purposes of analysis. Several different categories of age were assessed in preliminary analyses, but two were used in the final analysis: 1) 10-year age groups from 15–24 to 55–64 years, plus a category for age 65 and over; and 2) a dichotomous variable indicating whether the participant was aged 45 years or more (the maximum age was 81 years).

Socio-economic variables available included the age at which the participant left school, full-time employment status, housing tenure and private health insurance. Although information was collected on household income and highest educational qualifications, these variables were not included in the analysis due to a relatively large number of missing values.

The age at which the participant left school was collected using the following categories: still at school; never went to school; under 14 years; 14 years; 15 years; 16 years; 17 years; and 18 years or over. Based on the distribution of the data and the characteristics of the Australian educational system, data were combined to form three groups: less than 14 years or never went to school; age 14–15 years; and age 16 or more years or still at school.

Data on full-time employment were taken from one of a list of questions that were not mutually exclusive (e.g. working full-time, part-time student, home duties). For each item on the list, participants could respond "yes", "no" or "don't know/not sure". Participants were classified as being in full-time employment if they responded positively to the question on working full-time. Participants were classified as not being in full-time employment if they responded "no". Those who responded "don't know/not sure" were coded as missing.

Housing tenure was collected using the following categories relating to the participant's current place of residence: fully owned or being purchased by the participant or someone in the household; being rented; being occupied rent-free; and other: (please specify). Data were combined into two categories for analysis: owned or being purchased; and rented or other tenure.

Participants were considered to have private health insurance if they indicated they had any of the following: hospital cover only; extras cover only; both hospital and extras cover; or private health insurance of a type unknown to the participant. Participants were considered not to have private health insurance if they responded "no". Those who responded "don't know/not sure" were coded as missing.

Cultural variables included: whether the participant identifies with a clan, tribal or language group (yes/no); whether has non-Indigenous grandparents (yes/no/don't know); and whether identifies as a member or part of the Stolen Generations (yes/no/prefer not to answer). Responses of "don't know" or "prefer not to answer" were coded as missing. The Stolen Generations is a term referring to Indigenous people who were forcibly removed from their families as children by police or welfare officers as part of government assimilation policies [11]. The language for this potentially sensitive question was guided by the study's Indigenous Steering Group.

Other variables included in the analysis were the Indigenous status of the staff member who witnessed the consent form (Indigenous or non-Indigenous) and smoking status (current, former or never smoker).

### Baseline blood tests

A range of biochemical measures obtained from a fasting blood sample were also available. Collection and analysis methods have been described elsewhere [10]. These tests were part of the baseline health examination and were conducted for all participants who provided a fasting blood sample, regardless of whether they consented to long-term storage of excess samples.

### Statistical methods

**Figure 2:**
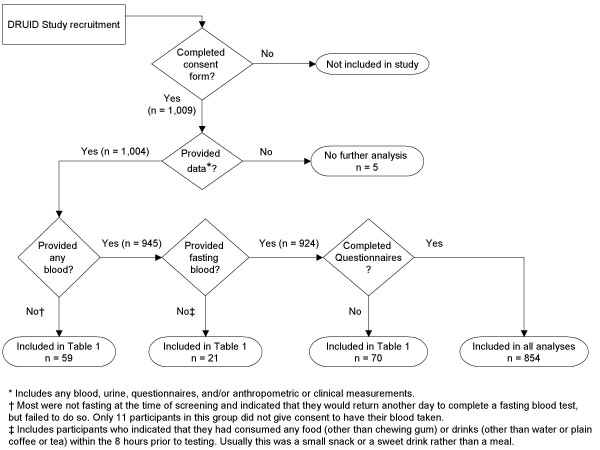
Participation in the DRUID Study.

All analyses were performed using Stata version 9 (Stata Corporation, College Station, TX). Pearson chi-squared tests were used to assess the statistical significance of comparisons of the proportion of participants giving consent to store blood with the proportions giving consent for other study components. This analysis included all those who completed a consent form and contributed any clinical, biochemical, anthropometric or questionnaire data (Figure 2). Simple and multiple logistic regression models were used to assess the associations between selected socio-economic, demographic and cultural variables and the dependent variable of interest (consent to store blood). Selection of variables for inclusion in the final model in Table 2 began by including those variables most strongly associated with the dependent variable, and subsequently adding and deleting variables based on changes in the fit of the model. Goodness of fit was assessed using likelihood-ratio tests to compare nested models [12]; a significance level of p < 0.10 was used for these tests. All variables excluded from the final model were only weakly associated with the dependent variable, with adjusted odds ratios between 0.8 and 1.25. Ordinary least squares regression models were used to estimate associations between consent to store blood and baseline blood test results, to assess the potential for selection bias in future studies using stored samples. As blood test results were not normally distributed, all values were natural log-transformed prior to regression modelling, and geometric rather than arithmetic means are presented. All logistic and linear regression analyses were limited to participants who provided a fasting blood sample and completed a questionnaire (Figure 2).

**Table 2:** Relationship of consent to store blood with selected socio-cultural, economic and demographic characteristics. Among 854 urban Indigenous Australian research participants who completed a questionnaire and provided a fasting blood sample in 2003–2005.

	% of total	% who agreed to long-term storage	Unadjusted model		Age-adjusted model		Final model†‡ (n = 770)	
			
			OR*	95% CI*	OR*	95% CI*	OR*	95% CI*
Sex								
Male	31.6	59.6	1.08	0.81, 1.45	1.10	0.82, 1.47		
Female	68.4	57.7	1.0	---	1.0	---		
Age group‡								
15–44	71.0	55.3	1.0	---	---		1.0	---
45–81	29.0	65.7	1.55	1.14, 2.11	---		1.49	1.04, 2.13
Indigenous group								
Aboriginal only	83.6	56.7	1.0	---	1.0	---	1.0	---
Torres Strait Islander only	5.8	70.0	1.78	0.96, 3.32	1.78	0.95, 3.33	1.85	0.96, 3.57
Both Aboriginal & Torres Strait Islander	10.5	64.4	1.38	0.88, 2.18	1.40	0.89, 2.22	1.59	0.97, 2.60
Age left school§								
< 14 years or never went	5.4	56.5	0.99	0.54, 1.81	0.77	0.41, 1.46	0.84	0.42, 1.69
14–15 years	22.7	63.5	1.33	0.95, 1.86	1.18	0.84, 1.68	1.49	1.02, 2.18
≥ 16 years or still at school	71.9	56.7	1.0	---	1.0	---	1.0	---
In full-time employment§								
Yes	43.7	57.5	0.93	0.71, 1.23	0.94	0.71, 1.24		
No	56.3	59.2	1.0	---	1.0	---		
Housing tenure§								
Owned or being purchased	42.5	61.3	1.24	0.94, 1.64	1.21	0.91, 1.60		
Rented or other tenure	57.5	56.1	1.0	---	1.0	---		
Private health insurance§								
Yes	24.5	65.8	1.54	1.09, 2.16	1.50	1.06, 2.11		
No	75.5	55.6	1.0	---	1.0	---		
Smoking status§								
Current smoker	43.5	52.2	1.0	---	1.0	---	1.0	---
Former smoker	24.2	60.8	1.42	1.00, 2.01	1.35	0.95, 1.91	1.24	0.85, 1.80
Never smoker	32.3	64.0	1.63	1.18, 2.24	1.59	1.15, 2.20	1.48	1.04, 2.10
Identifies with clan, tribal, language group§								
Yes	61.2	59.8	1.0	---	1.0	---		
No	38.8	56.7	0.88	0.66, 1.17	0.94	0.71, 1.26		
Has non-Indigenous grandparents§								
Yes	69.3	63.8	1.90	1.40, 2.58	1.98	1.45, 2.70	2.07	1.50, 2.85
No	30.7	48.1	1.0	---	1.0	---	1.0	---
Identifies as member of Stolen Generations§, ¶								
Yes	27.1	63.6	1.34	0.96, 1.86	1.27	0.91, 1.77		
No	72.9	56.7	1.0	---	1.0	---		
Staff member who witnessed consent is Indigenous§								
Yes	40.9	63.3	1.43	1.08, 1.89	1.36	1.02, 1.80	1.43	1.05, 1.94
No	59.1	54.8	1.0	---	1.0	---	1.0	---

* OR, odds ratio; CI, confidence interval. Both were obtained from a logistic regression model.

† Includes all factors for which an estimate is shown.

‡ Similar results were obtained using 10-year age groups.

§ N = 854 except as indicated: age left school (n = 846); full-time employment (n = 836); housing tenure (n = 844); private health insurance (n = 775); smoking status (n = 842); identifies with clan, tribal or language group (n = 828); has non-Indigenous grandparents (n = 785); identifies as a member of the Stolen Generations (n = 760); Staff member who witnessed the consent is Indigenous (n = 853).

¶ A term referring to Indigenous people who were forcibly removed from their families as children by police or welfare officers as part of government assimilation policies [11].

### Ethics approval

The study was approved by the Human Research Ethics Committee of the Northern Territory Department of Health & Community Services and Menzies School of Health Research. It was considered and approved by both the Aboriginal sub-committee, which has absolute right of veto, and by the main committee. The study's governance structure included an Indigenous Steering Group, as well as partnerships with key Indigenous organisations [10].

Funding sources played no role in the study design, in the collection, analysis and interpretation of the data, in the writing of the manuscript, or in the decision to submit the manuscript for publication. The corresponding author had full access to all the data in the study, and had final responsibility for the decision to submit for publication.

## Results

A total of 1,009 people completed a consent form. Of 1,004 participants who provided at least one measurement, just over half (54.8 percent) gave permission for long-term storage of excess samples. Of these, the majority (58.2 percent) indicated that they wanted to be contacted for their permission prior to any future use. Those who actually provided a blood sample (n = 945) were more than twice as likely to give consent for storage than were those who did not provide a sample following the consent process (56.5 percent versus 27.1 percent; *p *< 0.001).

Consent for long-term blood storage was less commonly given than consent for other aspects of the study (Table 1). Participants who agreed to allow their blood to be stored were also more likely to agree to other aspects of the study, such as allowing study staff to access information about them from various service providers and government agencies (Table 1).

Among those who provided a fasting blood sample and completed a questionnaire (n = 854), consent for long-term storage varied according to demographic, socioeconomic and cultural factors (Table 2). Consent was more likely among those aged 45 years or more (adjusted odds ratio (OR) = 1.5), never smokers (OR = 1.5), those who reported having at least one non-Indigenous grandparent (OR = 2.1), those whose consent form was witnessed by an Indigenous staff member (OR = 1.4), and those who left school at age 14 or 15 years (OR = 1.5) (Table 2). Participants who identified as Torres Strait Islander were more likely to agree to have their blood stored than those who identified as Aboriginal (OR = 1.9), although the 95 percent confidence interval was relatively wide. The relative odds of agreeing to storage were smaller (in the range 0.8–1.2) for other factors after adjusting for these variables. Results were similar using 10-year age groups rather than a dichotomous variable for age with categories of 15–44 and 45–81 years.

Consent for long-term storage was not associated with large differences in any biochemical measure examined, either before or after adjusting for 10-year age group and sex (Table 3). Compared with those who did not agree to storage, adjusted mean values for those who agreed to storage ranged from 9.7 percent lower for C-reactive protein to 8.7 percent higher for fasting insulin (among participants with diabetes), with most differences within ± 5 percent (Table 3). Among those who agreed to storage, prior blood test results were similar for those who did and did not want to be contacted prior to future use (data not shown).

**Table 3:** Relationship of consent to store blood samples with selected biochemical measurements. Among 854 urban Indigenous Australian research participants who completed a questionnaire and provided a fasting blood sample in 2003–2005.

		Did not agree to long-term storage (n = 356)		Agreed to long-term storage (n = 498)	
		
	n	Geometric mean	95% CI	Geometric mean	95% CI
Fasting glucose (mmol/L)					
All participants	854	5.5	5.3, 5.6	5.6	5.5, 5.7
Participants with diabetes†	149	8.7	7.8, 9.7	8.4	7.8, 9.0
Participants without diabetes†‡	548	5.0	4.9, 5.0	5.0	4.9, 5.0
Fasting insulin (mU/L)					
All participants	853	9.4	8.7, 10.2	9.8	9.1, 10.4
Participants with diabetes†	148	13.8	11.0, 17.3	15.0	12.8, 17.6
Participants without diabetes†‡	548	8.3	7.6, 9.1	8.0	7.5, 8.7
Total cholesterol (mmol/L)	849	4.9	4.8, 5.1	4.9	4.8, 5.0
HDL cholesterol (mmol/L)	849	1.1	1.1, 1.1	1.1	1.1, 1.2
LDL cholesterol (mmol/L)	813	3.0	2.9, 3.1	2.9	2.8, 3.0
Triglycerides (mmol/L)	849	1.5	1.4, 1.6	1.5	1.4, 1.5
Homocysteine (umol/L)	854	9.5	9.1, 9.9	9.4	9.1, 9.7
Fibrinogen (g/L)	829	3.7	3.5, 3.8	3.6	3.5, 3.8
Haemoglobin A1c (%)	843	5.5	5.4, 5.6	5.6	5.5, 5.7
C-reactive protein (mg/L)	848	3.2	2.8, 3.7	3.0	2.7, 3.4

		Estimated percent difference in the mean associated with consent to store blood sample*			
		Crude		Adjusted for age and sex	
		
		% difference	95% CI	% difference	95% CI

Fasting glucose (mmol/L)					
All participants		2.2	-1.4, 5.9	0.6	-2.8, 4.0
Participants with diabetes†		-4.0	-15.0, 8.3	-1.3	-12.8, 11.6
Participants without diabetes†‡		0.2	-1.3, 1.7	0.1	-1.4, 1.5
Fasting insulin (mU/L)					
All participants		3.6	-6.7, 14.9	1.7	-8.3, 12.8
Participants with diabetes†		9.1	-16.6, 42.7	8.7	-17.3, 42.8
Participants without diabetes†‡		-3.5	-14.2, 8.4	-4.0	-14.4, 7.8
Total cholesterol (mmol/L)		-1.7	-4.4, 1.2	-2.1	-4.7, 0.6
HDL cholesterol (mmol/L)		2.5	-1.4, 6.6	3.1	-0.7, 7.1
LDL cholesterol (mmol/L)		-2.1	-6.0, 2.1	-2.4	-6.3, 1.6
Triglycerides (mmol/L)		-3.6	-10.9, 4.3	-6.4	-12.9, 0.5
Homocysteine (umol/L)		-1.0	-5.8, 4.1	-2.7	-7.2, 2.0
Fibrinogen (g/L)		-0.1	-4.8, 4.8	-0.9	-5.5, 4.0
Haemoglobin A1c (%)		0.7	-1.9, 3.3	-0.7	-3.0, 1.6
C-reactive protein (mg/L)		-6.4	-21.3, 11.3	-9.7	-23.1, 6.1

* Calculated as 100(e^β ^-1), using the relevant regression coefficient from a linear regression model with the dependent variable measured on the log scale.

† Participants were classified as having diabetes if they met any of the following criteria: 1) fasting plasma glucose ≥ 7.0 mmol/L; 2) 2-hour post-glucose load plasma glucose ≥ 11.1 mmol/L; or 3) previously diagnosed as having diabetes and currently taking tablets and/or insulin for diabetes. Among participants who were not currently taking tablets and/or insulin for diabetes, impaired glucose tolerance (IGT) was considered present if fasting glucose was < 7.0 mmol/L and 2-hour glucose was ≥ 7.8 and < 11.1 mmol/L; impaired fasting glucose (IFG) was considered present if fasting glucose was ≥ 6.1 and < 7.0 mmol/L, and 2-hour glucose was less than 7.8 mmol/L.

‡ Excludes those with impaired fasting glucose or impaired glucose tolerance, or whose diabetic status could not be classified.

## Discussion

The results indicate that consent for long-term storage of excess blood samples for possible use in future studies was neither rare nor universal among these urban Aboriginal and Torres Strait Islander research participants. Consent for blood storage was strongly related to consent for other study components, although it was the component for which consent was least likely. It was associated with some but not all of the socio-demographic and cultural factors examined. Consent to store blood was not associated with substantial differences in previous blood test results, which suggests that any bias resulting from future use of stored samples is unlikely to be large, at least for analyses of similar substances.

A few studies have examined people's willingness to allow storage and use of their biological samples, with higher proportions among research participants and patients than among the general public. In one study of the general public, 43 percent of respondents indicated they would be willing both to donate blood for research "to find genes that affect people's health" and to have their blood stored for later use [[13]:20]. The impact on the results of an explicit reference to genetic research is unclear. In a study examining actual consent among research participants, 87 percent agreed to allow their samples to be used for future research. Similarly high proportions were seen for patients, family members and healthy volunteers, although a lower proportion (75 percent) of African American participants consented [14]. Other studies of research participants and clinical patients have reported similarly high levels of consent – much higher than in the present study – for future use of stored samples [15,16].

In the present study, cultural factors appeared to play a role in participants' decision-making. Although the exact nature of that role remains highly speculative, differing levels of trust – whether of institutions, processes, social groups and/or individuals – may be a common factor. For example, the greater likelihood of consent among those whose witness was Indigenous may reflect increased trust due to shared membership of a salient social group. Higher levels of consent among participants with non-Indigenous grandparents may reflect greater familiarity with mainstream health services, and a possible reduction in distrust of health-related institutions, including research organisations. Shavers and colleagues have suggested that lower research participation by African Americans is related to their lower levels of trust of medical research [4]. It is important to understand that "trust" is not a binary variable; it is possible to have varying degrees of trust, and to have trust in some things (or people) but not others. Trust in one dimension does not necessarily guarantee trust in other areas. In the present study, there were a number of interactions and processes in which the level of trust could be relevant, including: trust in the staff member administering the consent form; trust in the study staff collecting information, taking blood and/or taking clinical measurements; trust in the researchers named in the participant information materials; trust in the various institutions involved in the study; trust that blood samples and other participant information would be handled appropriately and respectfully; trust that participants' decisions about what should be done with excess samples would be carried out; and trust that stored samples would not be accessed in a way that might bring harm to participants. The results of this study suggest that there were varying degrees of trust relating to the storage and future use of excess blood samples among participants, with 45 percent choosing to have excess samples destroyed, 32 percent allowing storage but requiring permission for further use, and 23 percent agreeing to storage and future use of samples provided their use was approved by the study's Indigenous Steering Group. It is notable that, even among this last group, there is a requirement for community input and agreement. That is, participants were not asked to trust the researchers to decide unilaterally what could be done with excess samples, although they obviously needed to have sufficient trust in the integrity of the researchers to expect that they would follow the processes outlined in the consent form.

Participants who identified as Torres Strait Islanders were more likely to agree to long term blood storage than those who identified as Aboriginal, although the 95 percent confidence interval was relatively wide. Whether this reflects cultural differences is not known. It is important to note that Aboriginal Australians are an extremely heterogeneous group with respect to culture, language, and tribal affiliation, as well as social and economic circumstances. Unfortunately, it was not possible in this study to stratify the "Aboriginal" group into more meaningful sub-groups, in part because almost 4 in 10 participants indicated that they did not identify with a clan, tribal or language group.

Smokers were less likely to agree to storage than non-smokers. Indigenous Australians are about twice as likely to smoke as non-Indigenous Australians, and smoking is a major contributor to the ill health of Indigenous Australians [17]. Many Indigenous Australian groups used native tobacco and similar plants prior to contact with Europeans, and tobacco continues to play a ceremonial role in some areas, especially in Northern Australia [18,19]. It has been suggested that tobacco's role in ensuring social cohesion may be greater among Indigenous Australians than among other Australians [19], and that tobacco is now "embedded in the sociability and exchange of everyday life for thousands of Aborigines and Torres Strait Islanders" [[18]:120]. Thus smoking may represent a tension between maintaining a healthy lifestyle and enhancing one's social and cultural connectedness.

Blood has been described as carrying "a heavy cultural freight" [[20]:3011]; it is "inherently powerful" and "a topic of great cultural sensitivity" among at least some Indigenous Australian groups [[9]:25–26]. Although it is certain that some people did not participate in the study because they did not wish to have blood taken, it is not clear to what extent any blood-related non-participation was due to cultural considerations rather than other factors such as an aversion to needles or "bad veins".

Despite such potential sensitivities, the collection of blood was considered necessary to meet the main aims of the DRUID Study. By contrast, the long-term storage of blood was not essential, and the decision to proceed was a considered one. There are widespread perceptions that Indigenous Australians as a group have been "over-researched", but relatively little is known about the health and wellbeing of Indigenous people living in urban areas. The DRUID Study was, to our knowledge, the first large study of urban Indigenous adults ever undertaken in Australia, and the potential to answer future research questions without imposing additional burdens on members of the population was seen as attractive, albeit not without ethical challenges.

Decisions about requesting or giving consent for storage and later use of tissue samples must recognize the potential tension between two central ethical principles, namely justice and respect [21]. Justice includes maximising the usefulness of an individual's participation and sharing the research burden equitably. Respect includes supporting participants' ability to determine what happens with information collected from or about them. Maximising the scientific return for an individual's participation helps maximise community research benefits, but collecting biological materials may compromise participation, thereby potentially introducing bias and ultimately limiting a study's scientific value. In this study, we tried to take both justice and respect for autonomy into account by allowing retention of excess samples while giving participants a choice regarding their own samples. No information was available on how participants made their decision or on the criteria they used to determine whether or not to provide consent; further investigation in this area is warranted.

The ethical principle of respect for autonomy was clearly an important consideration in this study. Autonomy, defined as "the ability to make informed choices about what should be done and how to go about doing it" [[22]:53], is considered by Doyal and Gough to be one of two basic universal needs that must be met to enable individuals to "flourish" as human beings [22]. The other basic need is survival/physical health. Importantly, physical health and autonomy are considered to have equal priority and to apply across cultures. To the extent that health research improves physical health, participation in research could be viewed as being morally desirable. However, according to this framework, any such action could not be undertaken in a way that reduces autonomy without compromising the ability to meet an individual's basic needs.

A tension between justice and respect for autonomy is not necessarily inevitable. In the Australian National Health and Medical Research Council (NHMRC) guidelines on ethical conduct in Indigenous health research, these two principles are framed as complementary rather than competing. The NHMRC approach is based not on compliance with prescriptive rules, but on six core values: spirit and integrity; reciprocity; respect; equality; survival and protection; and responsibility [23]. These values are intended to guide researchers in establishing ethical relationships of trust with Indigenous communities. By facilitating the creation of meaningful opportunities for Aboriginal and Torres Strait Islander people to contribute to all facets of research, from identification of research priorities to development and implementation of research processes to analysis, interpretation and translation of data, this type of approach has the potential to maximise community benefit through both higher participation and higher scientific return. Such an approach shifts the focus away from the often paternalistic protection of "vulnerable" individuals towards a model of community partnership and inclusion [24,25]. In theory, cooperation and collaboration between communities and researchers can increase both the representation of "vulnerable" people in research and their access to the benefits of that research. In practice, most research involves unequal power relationships [26]; although this can make the development of true partnerships challenging, it is critical to find mutually agreeable ways to overcome such obstacles.

The present study was undertaken in a context of ongoing suspicion of the research enterprise on the part of many Indigenous Australians and their community leaders. Despite a shift over the past decade to greater Indigenous control of and involvement in research (as exemplified by the creation of the Indigenous-led Cooperative Research Centre for Aboriginal Health [27] and the NHMRC's Aboriginal and Torres Strait Islander Health Forum [28]), there continues to be a perception that Indigenous people are subjected to research that does not address their needs but which provides researchers with substantial benefits, such as prestige, fame, employment, higher degrees, etc. In such a context, researchers need to prove themselves worthy of trust over a sustained period if they are to build respectful and productive partnerships with communities. However, the requirements of this long-term process may compete with the research team's ability to meet the scientific aims of a particular project in the shorter term. In the present study, for example, we needed to balance autonomy against the risk of bias. Although we believed that it was "right" to have participants decide what would happen to their excess blood samples, we could not be sure whether such a process would result in a sufficient number of stored samples to be useful in the future, nor did we know whether consent for storage would be related to blood biochemistry. Although it appears that we have been reasonably fortunate in both respects, whether this is an indicator of the "correctness" of the original decision depends on one's underlying ethical approach.

As in other endeavours, what is "right" in research may be determined on the basis of a variety of factors, such as duties or rules, outcomes, the character of the people involved, or on ethical principles, such as justice, respect for autonomy, beneficence and non-maleficence [29]. Sponsors, researchers, community leaders and participants may operate using different ethical frameworks, and this may be especially relevant in a cross-cultural setting (although it could be argued that all research is cross-cultural to a greater or lesser degree). Even within research teams, more than one approach may be used concurrently, especially when multiple disciplines are involved, such as in the present study. The resolution of conflict can be difficult is such situations, even when the points of contention are clearly articulated, which is not always the case. When values are incommensurable – that is, when there is no common standard by which to evaluate options – it is not possible to make choices in a rational way [30], and the resulting compromises may be less than satisfactory to those involved.

The NHMRC guidelines on ethical conduct in Indigenous health research do not specifically address the issue of tissue storage and use, but they clearly indicate the responsibility of researchers to "do no harm to Aboriginal and Torres Strait Islander individuals or communities" [[23]:16]. This language reflects another important ethical consideration: the potential for harm – including stigma and discrimination – to non-participants and to social groups [7,8,31]. As part of working with the community to anticipate and address potential harms [31], any future use of stored samples must be considered and approved by the DRUID Study's Indigenous Steering Group, as well as relevant ethics committee(s), regardless of individual participants' prior consent.

The potential for harm is only one side of the ethical equation, however; it must be assessed against the potential for benefit, both to individuals and communities. As is the case with harms, however, different stakeholders, such as sponsors, researchers, community leaders and individual participants, may have different views about whether something is actually a benefit, and about the magnitude and relevance of any such benefit.

There has been considerable discussion in recent years about how to prevent exploitation in research, particularly in relation to clinical research undertaken in developing countries by researchers from developed countries. For example, a Fair Benefits Framework has been developed by researchers and ethicists as a means of assessing whether the distribution of benefits is "fair" in relation to the level of burdens borne [32]. Considerations about fairness and exploitation are relevant not only in the developing world but also for research in marginalised communities in developed countries, such as Indigenous peoples. The World Health Organisation has developed a guide to preparing research agreements between Indigenous peoples and research institutions; the aim of such agreements is to ensure transparency, an appropriate balancing of interests, and a shared understanding of a range of important issues, such rights, responsibilities and expectations [33]. Although we did not have a formal agreement in the present study, we tried to address what we – the researchers and the Indigenous Steering Group – considered to be the key issues of concern. For example, the Steering Group was involved in the development of study protocols and materials, in the recruitment and selection of staff, and in the development and implementation of the recruitment strategy; we developed agreed terms of reference for the Steering Group and the Chief Investigators Group and had cross-membership on the two groups to ensure communication and transparency; and we attempted to provide benefits at several levels, such as the provision of individual results and targeted health information for all participants, employment and training opportunities for local Indigenous people, and the provision of a part-time diabetes educator for the local Aboriginal community-controlled health service. Whether such putative benefits were sufficient to prevent exploitation in the context of this study remains an open question.

One of the challenges for researchers working with Indigenous communities is a commonly experienced lack of clarity about what the relevant "community" is, and who or what represents it. Indigenous communities are not homogeneous entities that speak with one voice. To the contrary, there are likely to be multiple perspectives and competing interests within a given community, and it can be difficult for researchers and research institutions to determine the appropriate body with which to negotiate. In Australia, as elsewhere, there are a large number of Indigenous community controlled organisations, such as Aboriginal Community Controlled Health Services, which have valuable experience in representing the often diverse and conflicting interests of the people they represent (although their representative status is not always uncontested) and in interacting with outside agents. As a result, such organisations are well-placed to be able to broker agreements between researchers and communities. However, because these organisations are often already under-resourced, it may be necessary for research institutions to provide practical support to enable them to undertake an active role in research brokerage [34].

## Conclusion

Consent for blood storage among these urban Indigenous research participants was neither rare nor universal and was associated with some but not all of the socio-demographic/cultural factors examined. Decisions about requesting or giving consent for storage and later use of tissue samples must recognize a number of important, and potentially competing, ethical and logistical considerations. Successfully negotiating the ethical maze is a challenging but critical element of research, especially that involving disadvantaged groups, to whom the benefits of research may not be obvious. Finding appropriate solutions can not be done by researchers in isolation, but must involve active community partnerships, building/maintaining relationships of trust, and real sharing of power and decision-making. Such an approach is time-consuming, but it is increasingly essential, not only on ethical grounds but on scientific grounds as well.

## Abbreviations

CI confidence interval

NHMRC Australian National Health and Medical Research Council

OR odds ratio

## Competing interests

The authors declare that they have no competing interests.

## Authors' contributions

JC and TD participated in the conception and design of the study. JC analysed the data and drafted the initial manuscript. JC and TD revised the manuscript for important intellectual content, and both authors read and approved the final manuscript.
